# Aortic Valve Leaflet Disruption: A Severe Complication of Impella 5.5

**DOI:** 10.7759/cureus.13235

**Published:** 2021-02-09

**Authors:** Alexander D Ghannam, Manabu Takebe, Taylor S Harmon, Scott Tatum, John Pirris

**Affiliations:** 1 Surgery, University of Florida College of Medicine, Jacksonville, USA; 2 Cardiothoracic Surgery, University of Florida College of Medicine, Jacksonville, USA; 3 Radiology, University of Florida College of Medicine, Jacksonville, USA

**Keywords:** aortic valve leaflet injury, aortic insufficiency, impella 5.5, coronary artery bypass grafting, heart failure, surgical aortic valve, high risk cabg, tavr, impella

## Abstract

A 73-year-old male with a history of severe coronary artery disease and prior coronary artery bypass grafting (CABG) presented with chest pain and elevated troponins. His workup revealed an ejection fraction of 15%, severe native coronary disease, as well as stenosis of his bypass grafts. He underwent a high-risk redo CABG with an Impella 5.5® (Abiomed, Danvers, MA) placement. The device was removed on postoperative day eight, at which time he went into cardiogenic shock from aortic valve leaflet injury. Given that he had no calcium deposits around the aortic valve annulus and severe aortic insufficiency, a multidisciplinary heart team decided he would be best served by a surgical aortic valve replacement. He was taken back to the operating room for a surgical aortic valve and intra-aortic balloon pump. His postoperative course was complicated by pneumonia, sepsis, and renal failure requiring continuous renal replacement therapy. He was discharged to a rehabilitation facility after 42 days. The following case encompasses the high morbidity risk of acute aortic valve insufficiency after Impella placement, never before documented in an Impella 5.5.

## Introduction

Impella® (Abiomed, Danvers, MA) is a microaxial flow catheter that provides mechanical circulatory support for the short term (less than or equal to 14 days), unloading the left ventricle in patients with cardiogenic shock [1,2]. Prior to 2019, the device came in two sizes (2.5 and 5.0). The 2.5 is a 7 Fr catheter able to generate 2.5 L/min of cardiac output, while the 5.0 device, a 9 Fr catheter, can generate up to 5.0 L/min of flow. The 2.5 Impella device is small enough to be placed percutaneously through the femoral artery, while the larger 5.0 device requires arteriotomy in either the axillary artery or directly into the aorta during cardiac surgery. Regardless of size, the device is positioned with the inflow end of the catheter in the left ventricle, across the aortic valve, and with the outflow end of the catheter in the ascending aorta. The catheter siphons blood from the left ventricle and ejects it into the ascending aorta providing adequate perfusion pressures in patients that are unable to generate enough cardiac output.

Impella has been established as an essential tool for multidisciplinary heart teams treating acute myocardial infarction with reduced ejection fraction (EF), high-risk coronary revascularization, left ventricular dysfunction, and cardiogenic shock [1]. By unloading the left ventricle, the device can decrease myocardial oxygen consumption during times of physiologic stress and ultimately reduce the damage to the myocardium [1,2]. In 2019, a new 5.5 version of the catheter was introduced to the market which was 9 Fr in size and also required surgical placement [2]. The most common complications are related to the vascular access site such as hematoma, pseudoaneurysm, or bleeding. Other severe risks associated with Impella use are stroke, myocardial infarction, acute renal dysfunction, hemolysis, thrombocytopenia, aortic valve injury, and death [2]. The following case demonstrates the first reported aortic valve injury with Impella 5.5.

This article was published as a preprint: Ghannam A, Takebe M, Tatum S, Pirris J. Aortic Valve Leaflet Disruption: A Severe Complication of Impella 5.5. Authorea, September 11, 2020; DOI: 10.22541/au.159985924.42825335

## Case presentation

A 73 year-old-male with a history of hypertension, hyperlipidemia, diabetes, chronic kidney disease, tobacco abuse, and coronary artery disease with previous coronary artery bypass grafting (CABG) in 1995, presented with chest pain, shortness of breath, and cough for three days. An initial evaluation in the emergency department revealed elevated troponins and an electrocardiogram with no ST elevations. Urgent cardiology evaluation and left heart catheterization revealed diffuse native coronary disease, patent left internal mammary to a diagonal artery, and two saphenous vein grafts with minimal blood flow. Transthoracic echocardiography (TTE) demonstrated an EF of 15% and no structural heart disease. An extensive workup revealed significant hibernating myocardium. A high-risk redo CABG was planned with the placement of Impella 5.5 for hemodynamic support postoperatively.

**Figure 1:**
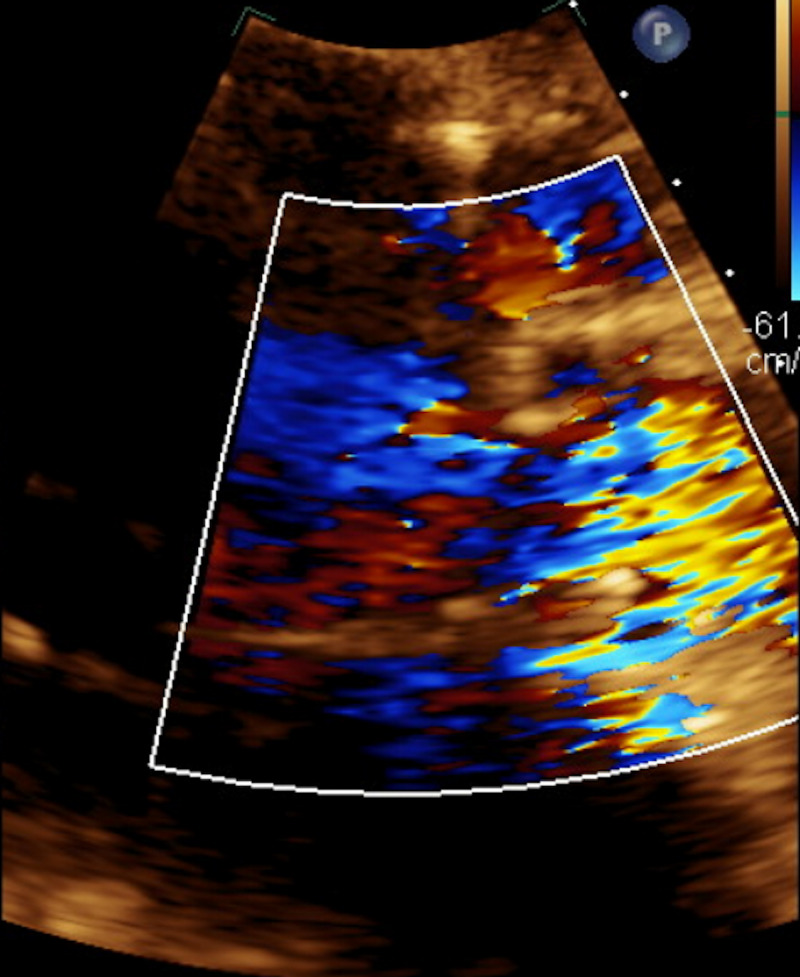
Severe Aortic Regurgitation The multispectral color doppler image demonstrates retrograde flow from the ascending aorta (in blue) consistent with aortic regurgitation.

**Figure 2:**
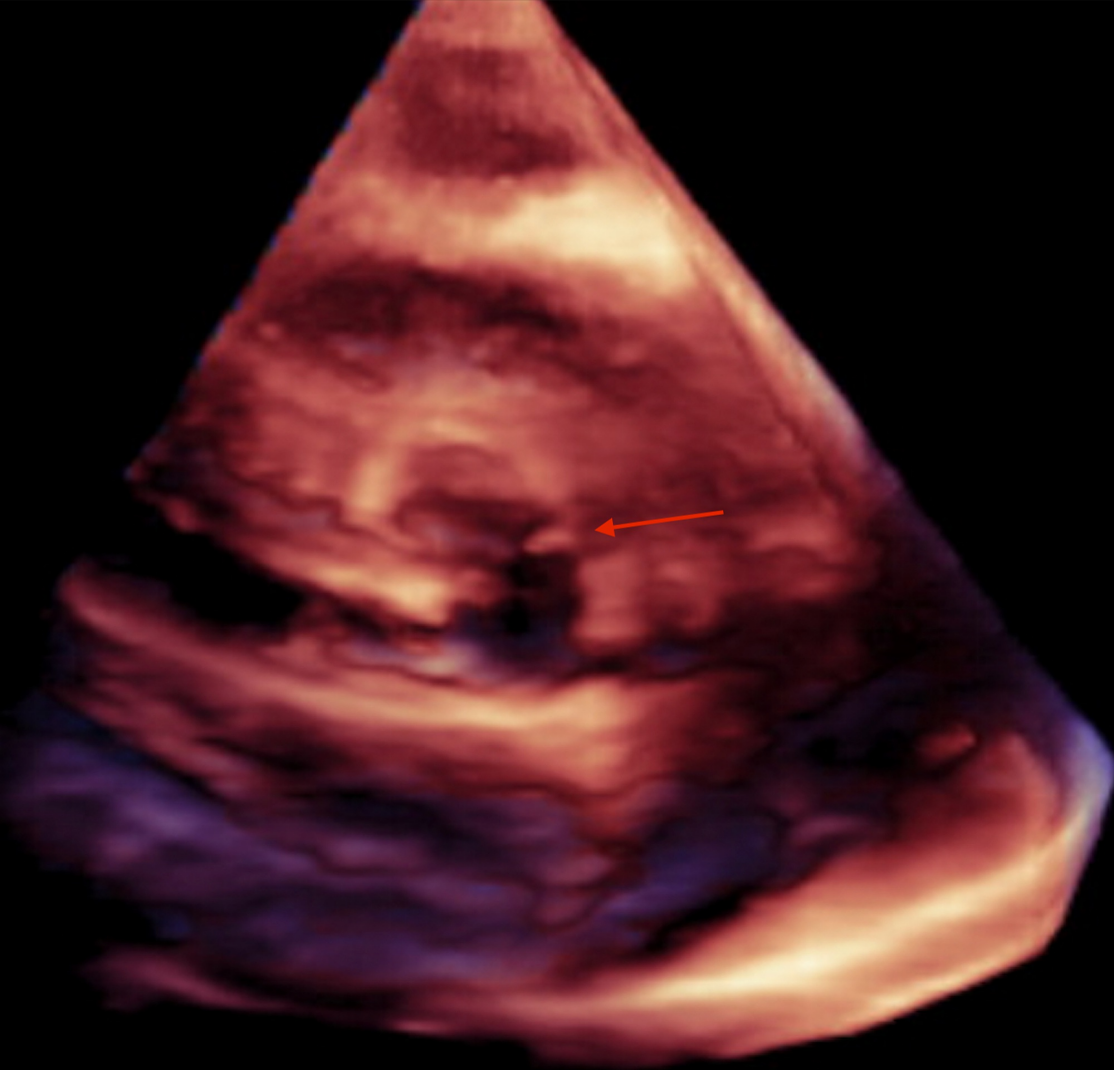
Aortic Valve Leaflet Laceration This is a three-dimensional echocardiographic image of the aortic valve with the arrow directed toward a tear of the left coronary leaflet.

The redo CABG was carried out in standard fashion; after the cross-clamp had been removed, the Impella was placed through a graft into the ascending aorta, without the use of a wire, while on full cardiopulmonary bypass. Once the device was in the correct position based on transesophageal echocardiography, the graft was tunneled out superior to the right clavicle. The patient's initial course was unremarkable and he was extubated on a postoperative day one. TTE on postoperative day three demonstrated an EF of 40% and appropriate positioning of the device. Daily chest radiographs also confirmed no significant device migration. On a postoperative day eight, he underwent Impella removal according to all safety standards in the manual [2]. Within a few hours of removal, he developed cardiogenic shock with a drop in his cardiac index from 3.1 L/min/m^2^ to 1.9 L/min/m^2^. Emergent repeat TTE demonstrated severe aortic regurgitation with a laceration of the left coronary cusp (Figures 1, 2). A multidisciplinary heart team was employed to discuss aortic valve replacement from a surgical or transcatheter approach. Due to a lack of calcium around the aortic valve annulus and concern for possible valve migration, the heart team decided to pursue a surgical aortic valve [3]. 

In the operating room, a 1 cm laceration of the left coronary cusp was identified. The native valve was excised and replaced with a 21 mm mechanical valve. The patient's postoperative course was complicated by pneumonia and severe kidney injury requiring temporary dialysis. He underwent tracheostomy and percutaneous gastrostomy tube placement and was eventually discharged to a rehabilitation facility after 42 days in the hospital.

## Discussion

While the Impella 5.5 is an integral tool in the armamentarium of heart teams caring for patients with cardiogenic shock, it is not without its pitfalls. Hong and Naseem describe mild to moderate malcoaptation of the aortic valve leaflets, identified while the Impella is in place or shortly after removal [4]. However, it is believed to be short-lived [4]. True iatrogenic aortic valve injury resulting in unrepairable damage to the aortic valve leaflets is a life-threatening complication of Impella in a patient population with severely compromised cardiac function. Treatment of aortic valve injury requires surgical valve replacement or conceivably transcatheter aortic valve replacement (TAVR) [5]. While the data for TAVR to treat aortic stenosis is well documented, there is significantly less evidence to support the use of transcatheter valves for aortic insufficiency [3]. While TAVR could have been used in this patient, our multidisciplinary heart team was concerned that due to the lack of calcium around his aortic valve, a transcatheter valve was at high risk for migration.

In the preceding case presented, it is unclear whether the valve was injured on insertion or removal, because of the known artifact on echocardiography [4]. It is also possible the aortic valve was injured during Impella support, however daily chest radiographs demonstrated no significant device migration. Although it is not required in the manual, we believe the best way to prevent aortic valve injury in the future is through the use of a guidewire and imaging (fluoroscopic and/or echocardiographic) guidance, regardless of the device insertion approach [2]. The use of live imaging can allow the operator to first ensure they cross the aortic valve with the wire safely and then to carefully advance the device across the aortic valve while feeling for resistance.

## Conclusions

Aortic valve injury resulting in aortic insufficiency and cardiogenic shock is a life-threatening complication of Impella use requiring surgical intervention. While Impella 5.5 is an excellent device for managing patients in cardiogenic shock, extreme care must be taken on insertion and removal to prevent aortic valve injury. Although not explicitly required in the manual, we believe inserting and removing the device should be done over a guidewire and with imaging guidance to prevent this complication in the future.
